# It matters what you see: Graphic media images of war and terror may amplify distress

**DOI:** 10.1073/pnas.2318465121

**Published:** 2024-07-05

**Authors:** E. Alison Holman, Dana Rose Garfin, Roxane Cohen Silver

**Affiliations:** ^a^Sue & Bill Gross School of Nursing, University of California, Irvine, CA 92697; ^b^Department of Psychological Science, University of California, Irvine, CA 92697; ^c^Department of Community Health Sciences, University of California, Los Angeles, CA 90095; ^d^Department of Medicine, University of California, Irvine, CA 92697; ^e^Department of Health, Society, and Behavior, Program in Public Health, University of California, Irvine, CA 92697; ^f^Department of Population Health and Disease Prevention, Program in Public Health, University of California, Irvine, CA 92697

**Keywords:** graphic images, media exposure, terrorism, mental health, collective trauma

## Abstract

Media exposure to graphic images of violence has proliferated in contemporary society, particularly with the advent of social media. Extensive exposure to media coverage immediately after the 9/11 attacks and the Boston Marathon bombings (BMB) was associated with more early traumatic stress symptoms; in fact, several hours of BMB-related daily media exposure was a stronger correlate of distress than being directly exposed to the bombings themselves. Researchers have replicated these findings across different traumatic events, extending this work to document that exposure to graphic images is independently and significantly associated with stress symptoms and poorer functioning. The media exposure-distress association also appears to be cyclical over time, with increased exposure predicting greater distress and greater distress predicting more media exposure following subsequent tragedies. The war in Israel and Gaza, which began on October 7, 2023, provides a current, real-time context to further explore these issues as journalists often share graphic images of death and destruction, making media-based graphic images once again ubiquitous and potentially challenging public well-being. For individuals sharing an identity with the victims or otherwise feeling emotionally connected to the Middle East, it may be difficult to avoid viewing these images. Through a review of research on the association between exposure to graphic images and public health, we discuss differing views on the societal implications of viewing such images and advocate for media literacy campaigns to educate the public to identify mis/disinformation and understand the risks of viewing and sharing graphic images with others.

Once again, the world is being rocked by terrorism and war, with horrific images from the war in Israel and Gaza ever present on our screens. Since the October 7, 2023, attack on Israel by Hamas, images of violence, death, destruction, hostages, and displaced persons have inundated the international 24-h news cycle, leading to debates and protests worldwide. As we saw soon after the start of the war in Ukraine in February 2022, gruesome pictures have proliferated on Instagram, TikTok, Facebook, X/Twitter, and YouTube, with sophisticated algorithms presenting more disturbing images to individuals as they continue to click (1). Because many people now carry smartphones equipped with powerful cameras that facilitate rapid distribution of graphic images across unregulated social media platforms, with a few quick swipes, these alarming images are easily accessed by both adults and children, etching horrific images into the minds of millions. While news coverage is essential to convey information to a populace during any crisis (2), the amount, content, and biases of such coverage must be balanced against the likely harmful effects of this media exposure on mental and physical health.

Following the September 11, 2001, terrorist attacks against the United States (9/11), the role of media exposure to collective trauma became a critical topic of psychological research as the public repeatedly witnessed images of the attack and its aftermath on television. Indeed, television exposure to stories and specific images of the attacks were associated with probable post-traumatic stress disorder and depression in a sample of New York residents up to 1 y post-9/11 (3–5). Similar effects were also seen across the United States (6). In the early aftermath of the attacks, our team found that 17% of a nationally representative sample of US residents living outside of New York City (N = 2,729) reported 9/11-related acute stress symptoms; high levels of post-traumatic stress symptoms (PTSS) remained evident in 6% of this national sample 6 mo later (7). These associations were persistent over time and predicted subsequent mental and physical health in a nationally representative sample of Americans: Hours of early television exposure to 9/11 coverage were associated with both PTSS and increased incidence of new onset physical health problems 2 to 3 y after 9/11 (8). The relevance of 9/11-related media images was even seen from a geographically distant site: Schoolchildren in London who witnessed the attacks only through television coverage reported heightened intrusive images, PTSS, and poor functioning 6 mo later (9).

Thus, the potential for media reports of terrorism to be linked to psychological distress across the populace is clear (10), with evidence suggesting the potential for long-term adverse physical health consequences with repeated media exposure (11). After the Boston Marathon bombings (BMB), our team conducted a more in-depth assessment of media exposure (12) using representative samples drawn from Boston, New York, and the remainder of the United States (*N* = 4,675). By this time, the post-9/11 media landscape had changed dramatically; social media sites now offered rapid access to stories, still photos, and videos of collective traumas as they occurred around the globe. Our post-BMB assessments included exposure to television, radio, print media (e.g., newspapers, magazines), online news sites, pictures and/or videos on social media, and updates on social media. We then examined the association between the number of daily hours of reported BMB-related media exposure across all sources in the week after the bombings and BMB-related acute stress symptoms. We found that more hours of BMB-related media exposure were directly associated with more acute stress symptoms (Fig. 1) (12). Importantly, we also showed that extensive media-based BMB exposure (averaging six or more hours/day) in the week following the BMB was a stronger predictor of BMB-related acute stress than being present at the site of the bombings or being in Boston that day (12). Thus, this “local” event became a collective trauma and many people exposed to the violence through both geographic proximity as well as media coverage reported symptoms of psychological distress.

**Fig. 1.**
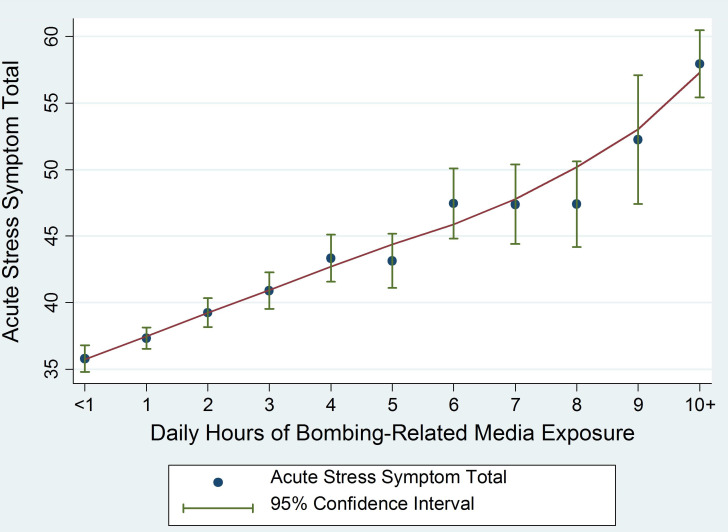
Acute stress symptom total by the number of hours per day of BMB media exposure in the week following the bombings. Reprinted from ref. 12.

To better understand how media is associated with mental and physical health outcomes, researchers have examined not merely the amount of exposure but the content of this exposure as well. The early research after 9/11 suggested that viewing certain images (e.g., persons falling from the World Trade Center) was likely to portend probable posttraumatic stress disorder and depression (3–5). Our team explored this possibility further in a study in which we followed our 9/11 sample longitudinally, collecting data during the 2003 Iraq war. We found exposure to specific images (United States/Allied POWs and dead Iraqi children) was uniquely associated with Iraq war-related acute stress symptoms and exposure to other images (soldiers in battle and dead United States/Allied soldiers) with 9/11-related PTSS 3 y after the attacks (8). In a subsequent analysis of our data following the BMB, we juxtaposed the amount of media exposure with frequency of seeing graphic (i.e., bloody) images. We found greater frequency of seeing such images independently predicted higher PTSS, fear of future terrorism, and poorer functioning 6 mo post-BMB, even after adjusting for how much BMB-related media exposure respondents reported (13). Taken together, these studies demonstrate that viewing graphic media images of violence and death is associated with detrimental physical and mental health outcomes.

Yet, each of these collective traumas does not exist in a vacuum. In the modern media environment, people view these media images in the context of those they have seen from previous events. After the BMB, among representative samples from the New York and Boston metropolitan areas, we found that those who had viewed prior collective traumas (i.e., 9/11, the Sandy Hook School shooting, Superstorm Sandy) live on traditional or social media reported higher BMB-related acute stress in the weeks following the BMB (14). We also followed a representative sample of US residents for several years, assessing their responses to multiple violent collective tragedies, including the 2016 Pulse nightclub shooting in Orlando, Florida, the largest mass shooting in US history at the time, where almost 50 patrons were killed and over 50 more were injured. We found engaging with collective trauma-related media was associated with a negative cycle of distress in which more media exposure to a collective trauma predicted more distress, more worry about the future, and ultimately more media exposure and greater distress following the next tragedy (Figs. 2 and 3) (15). Taken together, these findings demonstrate that both the amount and the content of media is associated with distress, as well as with a negative cycle that involves heightened distress, fear of future events, more media exposure, and more mental health impairments over time (15, 16).

**Fig. 2.**
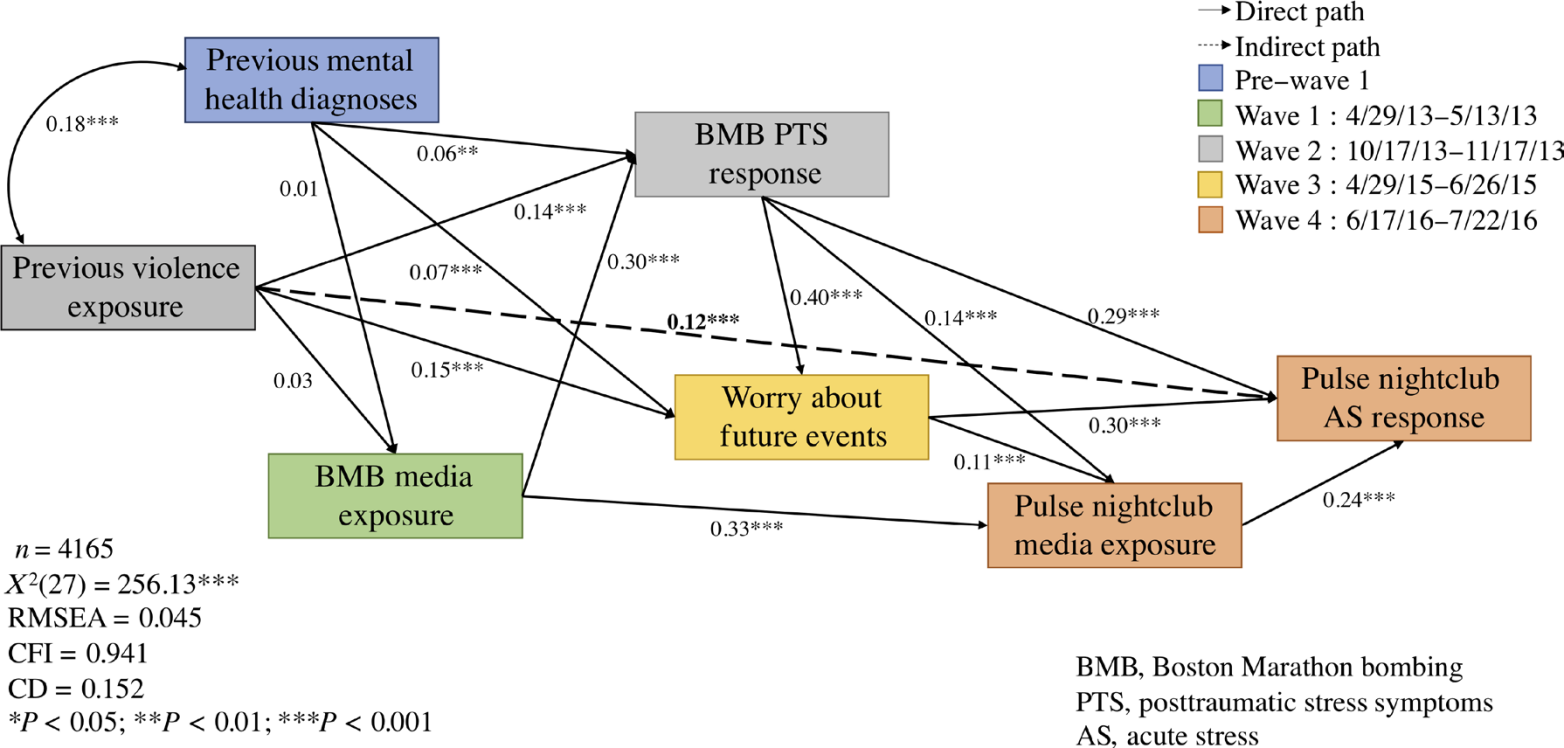
Path model predicting relationships between media exposures to collective trauma and psychological distress over time. Reprinted from ref. 15.

**Fig. 3.**
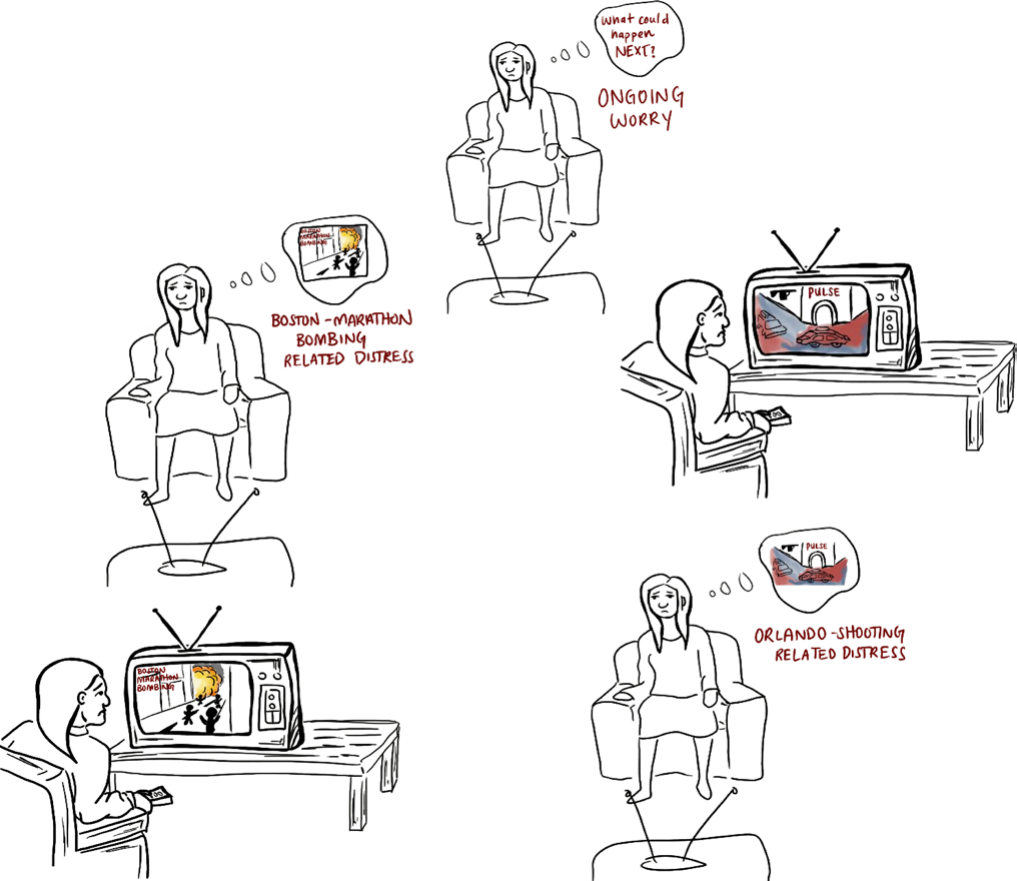
Cycle of media exposure to collective trauma, psychological distress, worry about the future, and more media exposure and distress following subsequent collective stressor (adapted from ref. 15), see https://youtu.be/cyQUHzc8XKc).

Even the initial shock of viewing graphic images once [and perhaps hearing the sounds of a violent tragedy; (17)] may contribute to mental health challenges on its own. Experimental data using functional MRI has evaluated the neural response to viewing traumatic imagery, finding such exposure activates fear circuitry in the brain (18), and that this activation is associated with subsequent “flashbacks” typically associated with traumatic stress responses following direct exposure. This is critical since the effects of repeatedly seeing media depictions of horror are cumulative and cyclical (14–16).

Of course, these findings beg the question of who is drawn to this media exposure and why it is difficult for some people to avoid engaging with media coverage of collective traumas. Several factors may make it challenging for people to refrain from viewing graphic images, even among those who suspect it is not psychologically beneficial to do so. Individuals who share an identity with the victims of a national or international tragedy or otherwise feel emotionally connected to a region and people who live there may be drawn to the news, feeling compelled to bear witness to the atrocities (19). We examined whether identifying with the victim of the violence strengthened the association between media exposure and viewers’ distress in a representative US sample surveyed 5 d after the Pulse nightclub massacre, which took place on Latin Night at a gay nightclub in Orlando, Florida. Results indicated that identifying as LGBTQ+ or Hispanic was positively associated with acute stress symptoms, which in turn was partly explained by exposure to more massacre-related media exposure. Those sharing both identities reported even greater acute stress (19).

Other studies have also focused on identifying what draws people to engage with media depictions of violence. Meta-analytic findings suggest that individuals who identify as men, and individuals with lower empathy, higher sensation seeking, and aggressiveness report more enjoyment from engaging with graphic media violence (20). It is also quite possible that pre-existing fear and anxiety draw people to view graphic images of violence, especially among individuals who have experienced violence in their past (21). In the aftermath of the widely disseminated ISIS beheading videos of journalists in 2014, we asked a representative sample of US residents if they had chosen to watch these videos. We found that those who watched a portion of at least one video reported having been previously exposed to violence during their lifetime and having a greater fear of future terrorism (21). War veterans with threat-related attentional biases measured using an eye-tracking paradigm reported more subsequent mental health symptoms than did veterans without this bias (22). Several reviews have documented that threat-related attentional biases have a small but robust association with mental health symptoms (23) but that this bias is not seen in individuals who are not anxious (24). These data suggest that individuals with pre-existing mental health ailments may be drawn to media images of graphic violence in part because of an attentional bias for threat-related stimuli. To address this potential confound in our prospective longitudinal studies, we have used samples for which pre-existing mental health status data are available, and the presence of preexisting mental health ailments are statistically controlled in our analyses. We have also documented robust associations between graphic media image exposure and distress among respondents who do not have pre-existing mental health problems. Thus, we suspect that the associations reported between graphic media image exposure and distress are not simply spuriously linked by threat-related attentional biases often seen in anxious individuals.

## Experimental Research on Media Exposure to Violence

Researchers have also used experimental methods to address the psychological impact of seeing violent images (see e.g., ref. 25). While this work has provided insights that are generally consistent with the correlational research, there are three issues that limit its generalizability to the current events on display in the media today. First, most of the experimental research on media violence has used fictional video game images of graphic violence as their stimulus (25). However, fictional and nonfictional images of graphic violence are created and used for very different purposes. Fictional violence is created as entertainment, and viewers know it is not real. This knowledge likely alters how viewers interpret and respond to the images and allows them to regulate their emotional response by focusing on specific features such as the production qualities of the depiction (cf. ref. 20). When viewing real graphic images of violence, this strategy is unavailable. Because experimental work on this topic has overwhelmingly used fictional images of graphic violence, it provides little information about how people respond to images of real-life graphic violence (also see ref. 21, where we offer a fuller discussion of this issue).

Second, to our knowledge, with a few exceptions (e.g., refs. 26–28), experimental studies have not used real-life images of graphic violence and they have largely examined outcomes quite different from those discussed above. One study showed bloody or gory images from the International Affective Pictures System (IAPS) (29); subjects were told they were real or were shown the same pictures with false Hollywood copyrights and told they were not real, and then were asked how negatively they felt about each of the images. Findings underscored the assertion that knowing images are fake decreases the negative affect one assigns to the images, especially for men, while women rated the real and fake images as equally negative. However, knowing how negative an image is scored is not the same as knowing how much distress a person experiences after seeing the image. A second study asked respondents to rate how pleasant and arousing IAPS images and images from the armed conflict in Columbia were using visual analog scales of happy/unhappy and sleepy/excited figures (27). Again, the affective valence assigned to an image is not equivalent to the experience of psychological distress evoked by an image. A third experimental study focused on how much anger, disgust, and moral sensitivity (26) participants felt after viewing scenes from a mass execution of Iraqis by ISIS, again not capturing the concept of psychological distress as measured in the aforementioned research. While the findings from each of these studies were generally consistent with prospective longitudinal results described above (e.g., real graphic images received more negative ratings), all these experimental studies used relatively small college student volunteer samples that rendered them vulnerable to selection biases and significantly limit the generalizability of the findings from this body of research.

## Media in the Context of War

The research described above clearly illustrates how widespread media coverage of tragedies can extend the boundaries of exposure far beyond those geographically proximal to the event (30). As the war in Israel and Gaza continues in the context of other escalating collective traumas (e.g., the ongoing war in Ukraine, mass violence events in the United States), we caution that there may be a precipitous risk to the public’s mental and physical health from engaging with graphic imagery emerging from the Middle East. To fully understand the association between media exposure to graphic violence and public health during times of war, it is critical to consider how media are used to help societies cope with war. During war, opposing parties are known to use media as propaganda to garner support for their positions (31), and war photography is often used to do so (32, 33). Importantly, media outlets may not critically assess the veracity of these claims before publishing them. As a result, the mediascape of war may include a great deal of misinformation and disinformation that can undermine the search for a peaceful solution (31, 34, 35). It can also create a media environment that can be readily manipulated to serve the interests of warring parties at the expense of public well-being. The expansive proliferation of graphic imagery in wartime is one of the ways this can happen (32). When this is done without fact-checking the authenticity of the images, it can contribute to the spread of misinformation, disinformation, and propaganda aimed at manipulating public sentiment about the war. Although seeing the suffering of people at the hands of an enemy may be distressing, it may also motivate support for the actions of political and military leaders. Indeed, this is one of the core purposes of wartime propaganda (36). Military leaders of warring parties understand this, and journalists are often embedded with military personnel to produce and distribute stories that depict a more positive image of the military (see refs. 37 and 38). In so doing they may generate support for their side by showing themselves in a more positive light to counter any propaganda advanced by their opponents (39).

In the context of war, media-based dissemination of propaganda aiming to incite anger against opponents and evoke sympathy for victims can spread war imagery far beyond the directly affected region. This has occurred throughout the reporting of the war in Israel and Gaza. At times, erroneous descriptions of graphic images have been shared to elicit sympathy (40). The Israeli government produced a graphic video showing the horror of the October 7th Hamas attacks that was screened to the Israeli Knesset, American politicians, and to an audience in Hollywood (41), in part to elicit support for Israeli military actions (42). Some of the images in this video were taken by Hamas via bodycams or mobile phones during their attack, while others were taken by Israeli emergency services and defense forces during recovery operations. At the same time, the widespread death and destruction in Gaza have been prominently shown in the media, with images of dead Palestinian civilians of all ages frequently appearing in social and mainstream media. Distributing such images during organized showings or on social media can inadvertently spread fear as well and may also amplify the psychological impact of the graphic images on the viewer.

## Debating the Value of Distributing Graphic Images

It is also important to note that as the approach to covering wars has evolved from individual media-based war photographers, to having media embedded with soldiers in the 2003 Iraq War, to soldiers wearing cameras to capture footage in real time, significant disagreement has emerged about the value and impact of sharing these images with the public. While social commentators have pointed out the danger of their potential to “anesthetize” the viewer (43), they have also asked whether we are obliged to see these images so we can understand the horror of war and other forms of human barbarism, and perhaps be motivated to do something about it (44, 45). After 9/11, media communication experts argued that seeing graphic images of violence, especially collective violence like that of the Holocaust or 9/11, helps both individuals and the collective heal as we bear witness to the trauma (46). People who closely identify with the victims may also feel the need to view and share images to make sure people are aware of what is happening and that their side of the story is being heard. They may feel obliged to share images with the hope that the images galvanize support for ending the violence. Both Hamas leadership and Israeli Defense Force leaders have acknowledged that graphic imagery was shared so the world would see the atrocities committed by the other side, to embolden their fighters, and garner public support for their own actions (47, 48).

Researchers have discussed the importance of seeing graphic images to strengthen moral sensitivity, shape public opinion, and facilitate their policy goals (fighting war, halting immigration) (26, 49). Indeed, widespread sharing of graphic images may increase anger and disgust that lead to greater moral sensitivity and individuals’ desire for government action (26) or contribute to policy debate in governments (50). For example, in response to the ongoing war in Israel and Gaza, the US State Department cautions about the possibility of retaliatory attacks in the United States and elsewhere (51), necessitating a vigilant population that supports counterterrorism efforts. Relatedly, some commentators have gone so far as to argue that we live in “the golden age of brutal voyeurism” (47) that essentially promotes the sharing of graphic war-related imagery to mobilize public outrage against an enemy, to satisfy their curiosity, or to score political points. However, disseminating these images to achieve these political aims fails to consider the potential for a tremendous emotional impact on the viewer. It also risks spreading misinformation or disinformation used by warring parties seeking to spread their message widely, gain sympathy, and intimidate their opponents (52). Moreover, there is no rigorous empirical evidence to support the assumption that seeing graphic images motivates prosocial behaviors among the populace, and there is evidence instead to suggest that seeing graphic images is associated with more mental distress and poorer functioning (13). Given the state of the field, much more work needs to be done to fully understand and inform the debate on the value of viewing graphic images (See refs. 53–55).

## Future Research Agenda

The research conducted thus far on the association between graphic imagery and public well-being has many strengths: the use of regionally or nationally representative samples (3–8, 12); some studies have prospective longitudinal designs with high retention rates over time, respondent mental health reported prior to the collective trauma, and multiple follow-up assessments conducted over years following the event (8, 15). Nonetheless, these studies are correlational, making it impossible to confirm a cause–effect relationship linking exposure to graphic images and health outcomes.

We need more expansive, robust research addressing media exposure to collective traumas (Table 1). For example, we need to better understand peoples’ motivations to engage with graphic images of real-world violence when they suspect doing so may not be healthy. What prevents people from stopping themselves from viewing this content? Perhaps the uncertainty created by collective trauma may encourage more media use in its aftermath (56), which contributes to people becoming caught in a cycle of distress. What are the best strategies for educating the public about the potential dangers of watching and spreading graphic imagery? Studies should address whether sharing an identity with the victims motivates people to behave in prosocial ways (57), whether trust in media sources is associated with these responses (58), and the mechanisms underlying the potential impact of still images, videos, sounds, and written depictions of graphic violence. The advent of AI-generated deep fakes raises further questions about how awareness of disinformation is affecting responses to graphic content. Can people distinguish between real and fictional AI-generated images? As the media landscape rapidly evolves, researchers must stay apprised of the ways technology is being used to further the aims of violent individuals and groups at the expense of the public’s mental and physical health (see Table 1 for other important research questions).

**Table 1.** Important research questions for exploring associations between media exposure to graphic images and mental/physical health

Question	Sample research questions
Why do people view graphic media, even when they suspect doing so may be unhealthy?	● What personal traits/experiences are associated with viewing more graphic imagery (e.g., identification with the victim, political affiliation/ideology, empathy/compassion, mental health status, prior exposure to violence, tolerance of uncertainty, cumulative prior trauma exposure)? ● What qualities of collective traumas are associated with viewing graphic imagery (e.g., uncertainty, number of fatalities, proximity, similarity to one’s own prior experience)? ● What contextual factors (e.g., political polarization, sociocultural milieu) are associated with individuals’ likelihood of viewing graphic imagery? ● What are the best strategies to help people limit their exposure to or spreading of graphic images?
Who is most vulnerable to the mental and physical health symptoms associated with viewing graphic images?	● What are the demographic correlates of people who experience mental and physical health symptoms after viewing graphic images? ● What personal traits/experiences are associated with vulnerability to experiencing health symptoms after viewing graphic imagery?
What are the mechanisms linking exposure to graphic images with mental and physical health?	● What physiologic systems/brain regions are activated when viewing graphic imagery? ● What is the connection, if any, between these systems/regions and mental and physical health?
What specific characteristics of media exposure to graphic violence are associated with mental and physical health symptoms?	● Are still images, videos, audio, and/or written depictions of graphic violence all associated with mental and physical health symptoms? If not, which format is most strongly associated with negative outcomes? ● What specific content in the imagery is most strongly associated with mental and physical health symptoms (e.g., blood, military action, human death)? ● Does repetitive exposure to graphic imagery have a stronger association with mental and physical health symptoms than single exposure? ● Does trust in the media buffer the association between graphic image exposure and mental and physical health symptoms?
What risk communication/educational strategies are most effective for preventing exposure to graphic images of violence?	● What is the best approach to educate individuals and the public at large about the risk of exposure to graphic images? ● What educational strategies are most effective in mitigating the risk associated with viewing graphic images of violence? ● What media-focused interventions can effectively limit how (e.g., automatic vs. with a warning), how much (e.g., frequency, repetition), and how graphic the media images are (e.g., with vs. without images of hurt people)?
Do people habituate to seeing graphic images or are there sensitization effects? Do both exist depending on the circumstances or person?	● What is the nature of the association between qualities of graphic images and habituation and/or sensitization to seeing them? ● If some individuals habituate and others are sensitized by exposure to graphic images, what are the risk and protective factors for habituation and for sensitization? ● What are the long-term health correlates of habituation and sensitization to graphic media images?
Are there positive outcomes associated with seeing graphic images of violence?	● Is exposure to graphic images associated with compassionate action to help the victims (e.g., prosocial behaviors); political actions to reduce the likelihood of future violence (e.g., protecting victims of domestic violence, gun control laws); community-based actions to reduce the risk of neighborhood violence; etc.? ● Does identification with the victims motivate people to engage in prosocial behaviors to help victims?
How will AI affect exposure and response to graphic images of violence?	● Can the public distinguish between AI-generated graphic disinformation and real events? ● Do people respond similarly to real-life vs. AI-generated images? ● How might technology be used to advance the aims of violent people/groups? Alternatively, can technology help promote public well-being when these images are seen?

To address these questions, methodologically rigorous experimental research can shed light on the directionality of the associations identified in correlational work. Ideally, such experiments would be conducted using ecologically valid methods that facilitate the translation of their findings to real world events as they occur, while being sensitive to the ethical challenges of exposing individuals to graphic imagery. Experiments conducted in the context of prospective longitudinal studies using representative samples would be ecologically valid, offer more generalizable findings, while reducing the risk of selection bias and other threats to the validity of the studies. Such studies could address questions about the efficacy of risk communication strategies or trigger warning framings or positive responses to seeing these images. To address potential ethical concerns, researchers should screen out participants who have ongoing mental health diagnoses or have experienced mental health symptomatology. In sum, creative approaches should be developed to allow experimental testing to determine whether graphic media exposure is causally linked with psychological distress and to examine the mechanisms underlying this link and the media qualities most closely linked with distress.

## A Call to Action

A benefit of living in the United States is appreciating the importance of freedom of the press. Of course, we agree that it is critical not to infringe on our freedom to distribute, see, or hear information. Moreover, it can be difficult for journalists and individuals to draw the line between visuals that convey the extent of atrocities and those that increase risk for psychological and even physical harm. In such a media environment, some might argue that we should ban public sharing of graphic imagery. However, this response ignores the potential benefits to society of allowing people to record and share violent events occurring in their communities. Indeed, having live video from some events—even graphic violence—can be essential for holding individuals accountable for their actions. For example, the video of George Floyd’s murder held the officers responsible and was a catalyst for a societal conversation about police brutality and inspired reforms across the country. Since the advent of social media and smartphones that allow us to record events as they occur, the public has become increasingly aware of police violence in communities of color. This growing awareness may have contributed to the widespread protests in the wake of George Floyd’s death and a growing public willingness to speak up against injustice.

However, widespread public viewing of real-life graphic violence, especially when it comes without warning, can make it hard for people not to see images they find distressing. Indeed, supermarkets, gas stations, and airports are just a few of the venues where one can readily see news stories with graphic images displayed. This automatic viewing could be difficult for individuals who have a threat-related attentional bias since their reflexive response to threatening images would be to look at them, and perhaps linger on them (see refs. 23 and 24). These kinds of exposures are also especially challenging because the element of surprise may contribute to the secondary trauma and distress experienced by the viewer (59).

The difficulty with regulating this behavior is that media companies benefit financially from drawing the attention of viewers, and this motivates them to use eye-catching imagery toward that end (60). Any attempt to regulate their content would undoubtedly be met with numerous legal challenges (e.g., First Amendment Free Speech Rights). That said, the Federal Communication Commission did enact a ban on obscene, indecent, and profane language from 6 AM to 10 PM to protect children, and this was instituted after legal battles were adjudicated by the US Supreme Court in 1978 (61). An argument could be made to ban graphic images on public media screens as viewing them may be associated with mental distress. However, such efforts may be met with resistance from groups representing the victims of mass violence, some of whom feel strongly that these images must be seen to motivate people to end gun violence and strengthen our humanity (see also refs. 59 and 62). That said, if seeing graphic images prevents further violence, we should have seen reductions in violence by now, given how frequently these images are shared on social media. Moreover, banning some images may produce reactance (cf. ref. 63) and paradoxically lead to greater public interest in seeing them. In fact, such bans have recently been linked with more toxic, antisocial behaviors in some online settings (64). Thus, regulating media exposures to real-life graphic images is an important topic, but one fraught with concerns. While we benefit from living in a society that values and protects First Amendment rights, the boundaries for harmful use of these rights are constantly in debate (e.g., allowing or prohibiting anti-vaccine misinformation on social media), with newsrooms regularly debating whether sharing specific images would be beneficial or harmful to the public. As the media have a financial conflict of interest related to sharing these images, to maximize First Amendment protections, perhaps it would be best to empower the public with the knowledge they need to make appropriate decisions for themselves.

Toward that end, an alternative approach, such as one advocated by experts on adolescent social media use, is to develop and implement widespread media literacy campaigns to educate the public about the pros, cons, and risks of media exposures (65). This would naturally include building awareness of the risks of seeing graphic imagery and thus allowing people to make a rational choice about whether to view such images. Over time, this may lead to lower demand to see these images, thereby reducing their prevalence in the media landscape. In the age of AI, a very important component of a media literacy campaign would be to educate the public about how to identify misinformation, disinformation, deep fakes, and other AI-generated fabrications and the potential consequences of sharing them. The public must be able to distinguish fact from fiction, as AI-generated disinformation poses a unique danger to public health and well-being when combined with social media algorithms and the speed with which people respond on these platforms. Such programs have been successfully implemented around the world (66, 67). To facilitate this process, mass media could use their vast resources to help educate the public on identifying disinformation by doing educational public service announcements that take people through the steps of fact-checking stories so that they have the knowledge to assess what is and is not real. Programs could be implemented in schools, and as individuals we can become advocates for media literacy campaigns with local politicians and community leaders.

## Conclusion

A few decades ago, the Hamas massacre and hostage taking in Israel and the death and destruction in Gaza that has followed might have remained a more localized tragedy. Many living elsewhere might have expressed sadness about lives cut short too soon, wrung their hands about a tragedy across the world, and moved on to the next story that filled their airwaves. Over 20 years ago, news editors across the United States decided to stop showing pictures of individuals falling from the World Trade Center during the 9/11 tragedy out of concern for the potential negative effects of exposing the population to such imagery. However, things are different in 2024. Like decisions made following the Tree of Life Synagogue shootings, Uvalde, Texas massacre, and the war in Ukraine, media outlets may now be debating whether—and how often—they should play sounds of people screaming or bombs dropping from the sky or show videos of the murder of civilians. Given the context of the research mentioned above, we can no longer wait for news editors and social media organizations to stop this distribution. Given the current media landscape, it is critical that we do it ourselves. In fact, choosing not to watch or distribute these images prevents the spread of terror and intimidation. It is an act of compassion for oneself, the victims, and one’s community. It is a socially responsible choice.

While seeing the recent horrors in Israel and Gaza may be unavoidable for those who are living through it, we can choose not to spread the horror while still acknowledging the difficulties experienced by innocent civilians. We would argue that we should turn off newscasts that loop graphic videos, avoid sites that splash gruesome pictures on their pages, and resist the desire to search for images online (21). If others post graphic videos, we should refrain from watching and sharing them. Indeed, it is critical to reduce these exposures to protect our health. This does not mean avoiding the news altogether, but rather scheduling when and how we engage with it. We can learn how to bear witness without doomscrolling and repeatedly exposing ourselves and others to graphic violence.

Finally, we must remember the broader context in which these events are occurring. The Hamas attacks on Israel and the ongoing war are not just a collective trauma—they have also been experienced through the lens of intergenerational, historical traumas experienced by Jewish, Israeli, and Palestinian people globally. To minimize these historical triggers, we must choose not to spread the fear and anxiety. We must remember that engaging with the graphic images plays into the hands of those leaders who are deliberately using the media to circulate these images with the aim of manipulating public opinion and emotion. Moreover, the spread of these images may place vulnerable individuals—those who are emotionally tied to the events, who have experienced violence in the past, or have mental health challenges—at greater risk for harm. We need to break the cycle of violence by acting with compassion to monitor our media exposure, avoiding the images, and refusing to spread the horror with the click of a button.

## Data Availability

There are no data underlying this work.

## References

[1] M. Cinelli, G. De Francisci Morales, A. Galeazzi, W. Quattrociocchi, M. Starnini, The echo chamber effect on social media. Proc. Natl. Acad. Sci. U.S.A. **118**, e2023301118 (2021). 33622786 10.1073/pnas.2023301118 PMC7936330

[2] S. J. Ball-Rokeach, M. L. DeFleur, A dependency model of mass-media effects. Commun. Res. **3**, 3–21 (1976).

[3] J. Ahern, S. Galea, H. Resnick, D. Vlahov, Television images and probable posttraumatic stress disorder after September 11: The role of background characteristics, event exposures, and perievent panic. J. Nerv. Ment. Dis. **192**, 217–226 (2004). 15091303 10.1097/01.nmd.0000116465.99830.ca

[4] J. Ahern , Television images and psychological symptoms after the September 11 terrorist attacks. Psychiatry **65**, 289–300 (2002). 12530330 10.1521/psyc.65.4.289.20240

[5] K. T. Bernstein , Television watching and the risk of incident probable posttraumatic stress disorder: A prospective evaluation. J. Nerv. Ment. Dis. **195**, 41–47 (2007). 17220738 10.1097/01.nmd.0000244784.36745.a5

[6] W. E. Schlenger , Psychological reactions to terrorist attacks: Findings from the National Study of Americans’ Reactions to September 11. JAMA **288**, 581–588 (2002). 12150669 10.1001/jama.288.5.581

[7] R. C. Silver , Nationwide longitudinal study of psychological responses to September 11. JAMA **288**, 1235–1244 (2002). 12215130 10.1001/jama.288.10.1235

[8] R. C. Silver ., Mental- and physical-health effects of acute exposure to media images of the September 11, 2001, attacks and the Iraq War. Psychol. Sci. **24**, 1623–1634 (2013). 23907546 10.1177/0956797612460406

[9] E. A. Holmes, C. Creswell, C. T. G. O’Connor, Posttraumatic stress symptoms in London school children following September 11, 2001: An exploratory investigation of peri-traumatic reactions and intrusive imagery. J. Behav. Ther. Exper. Psych. **38**, 474–490 (2007). 10.1016/j.jbtep.2007.10.003 18023425

[10] T. L. Hopwood, N. S. Schutte, Psychological outcomes in reaction to media exposure to disasters and large-scale violence: A meta-analysis. Psychol. Viol. **7**, 316–327 (2017).

[11] E. A. Holman , Terrorism, acute stress, and cardiovascular health: A 3-year national study following the September 11^th^ attacks. Arch. Gen. Psych. **65**, 73–80 (2008). 10.1001/archgenpsychiatry.2007.6 18180431

[12] E. A. Holman, D. R. Garfin, R. C. Silver, Media’s role in broadcasting acute stress following the Boston Marathon bombings. Proc. Natl. Acad. Sci. U.S.A. **111**, 93–98 (2014). 24324161 10.1073/pnas.1316265110 PMC3890785

[13] E. A. Holman, D. R. Garfin, P. Lubens, R. C. Silver, Media exposure to collective trauma, mental health, and functioning: Does it matter what you see? Clin. Psychol. Sci. **8**, 111–124 (2020).

[14] D. R. Garfin, E. A. Holman, R. C. Silver, Cumulative exposure to prior collective trauma and acute stress responses to the Boston Marathon bombings. Psychol. Sci. **26**, 675–683 (2015). 25896419 10.1177/0956797614561043

[15] R. R. Thompson, N. M. Jones, E. A. Holman, R. C. Silver, Media exposure to mass violence events can fuel a cycle of distress. Sci. Advan. **5**, eaav3502 (2019). 31001584 10.1126/sciadv.aav3502 PMC6469939

[16] E. A. Holman, D. R. Garfin, P. Lubens, R. C. Silver, “Hours vs. images: Understanding how media-based collective trauma exposure is linked to psychological wellbeing” in How Does Media Coverage of Traumatic Events Impact the Populace and Those Who Report It? Evidence from Survey and Experimental Research. Symposium conducted at the annual meeting of the International Society for Traumatic Stress Studies, D. R. Garfin, Chair (International Society for Traumatic Stress Studies, Dallas, TX, 2016). November 2016.

[17] H. A. Turner , Gun violence exposure and posttraumatic symptoms among children and youth. J. Traum. Stress **32**, 881–889 (2019). 10.1002/jts.22466 31833114

[18] C. Bourne, C. E. Mackay, E. A. Holmes, The neural basis of flashback formation: The impact of viewing trauma. Psychol. Med. **43**, 1521–1532 (2013). 23171530 10.1017/S0033291712002358 PMC3806039

[19] D. P. Relihan, N. M. Jones, E. A. Holman, R. C. Silver, Shared social identity and media transmission of trauma. Sci. Rep. **13**, 11609 (2023). 37463937 10.1038/s41598-023-33898-2 PMC10354080

[20] C. A. Hoffner, K. J. Levine, Enjoyment of mediated fright and violence: A meta-analysis. Media Psychol. **7**, 207–237 (2005).

[21] S. Redmond, N. M. Jones, E. A. Holman, R. C. Silver, Who watches an ISIS beheading—And why. Amer. Psychol. **74**, 555–568 (2019). 30802079 10.1037/amp0000438

[22] C. G. Beevers, H.-J. Lee, T. T. Wells, A. J. Ellis, M. J. Telch, Association of predeployment gaze bias for emotion stimuli with later symptoms of PTSD and depression in soldiers deployed in Iraq. Am. J. Psychiatry **168**, 735–741 (2011). 21454916 10.1176/appi.ajp.2011.10091309

[23] K. Clauss, J. Y. Gorday, J. R. Bardeen, Eye tracking evidence of threat-related attentional bias in anxiety- and fear-related disorders: A systematic review and meta-analysis. Clin. Psychol. Rev. **93**, 102142 (2022). 35279537 10.1016/j.cpr.2022.102142

[24] Y. Bar-Haim, D. Lamy, L. Pergamin, M. J. Bakermans-Kranenburg, M. H. van IJzendoorn, Threat-related attentional bias in anxious and nonanxious individuals: A meta-analytic study. Psychol. Bull. **133**, 1–24 (2007). 17201568 10.1037/0033-2909.133.1.1

[25] N. L. Carnagey, C. A. Anderson, B. J. Bushman, The effect of video game violence on physiological desensitization to real-life violence. J. Exper. Soc. Psych. **43**, 489–496 (2007).

[26] M. Grizzard , Graphic violence as moral motivator: The effects of graphically violent content in news. Mass Commun. Soc. **20**, 763–783 (2017).

[27] C. Hurtado-Parrado , Emotional response to pictures of the armed conflict in Colombia. Peace Conflict: J. Peace Psychol. **26**, 202–212 (2020).

[28] M. J. Kobach, A. J. Weaver, Gender and empathy differences in negative reactions to fictionalized and real violent images. Commun. Rep. **25**, 51–61 (2012).

[29] P. Lang, M. M. Bradley, “The International Affective Picture System (IAPS) in the study of emotion and attention” in Handbook of Emotion Elicitation and Assessment, J. A. Coan, J. J. Allen, Eds. (Oxford University, 2007), pp. 70–73.

[30] P. Vasterman, C. J. Yzermans, A. J. E. Dirkzwager, The role of the media and media hypes in the aftermath of disasters. Epidemiol. Rev. **27**, 107–114 (2005). 15958431 10.1093/epirev/mxi002

[31] S. Lewandowsky, W. G. Stritzke, A. M. Freund, K. Oberauer, J. I. Krueger, Misinformation, disinformation, and violent conflict: From Iraq and the “War on Terror” to future threats to peace. Amer. Psychol. **68**, 487–501 (2013). 24128313 10.1037/a0034515

[32] M. Griffin, Picturing America’s ‘War on Terrorism’ in Afghanistan and Iraq: Photographic motifs as news frames. Journalism **5**, 381–402 (2004).

[33] P. Lubens, E. A. Holman, “Media and disaster: Exploring the unintended consequences of disaster-related media coverage for public well-being” in Textbook of Disaster Psychiatry, R. Ursano, Ed. (Cambridge University Press, ed. 2, 2017), pp. 181–192.

[34] T. Hsu, S. Frenkel, From opposite sides of war, the hunt for elusive facts. New York Times, 25 January 2024. https://www.nytimes.com/2024/01/25/business/media/misinformation-fact-checking-israel-hamas.html?login=smartlock&auth=login-smartlock. Accessed 18 November 2023.

[35] N. Adams , Israel-Hamas War misinformation tracking center: 111 myths about the conflict and counting. NewsGuard, 21 March 2024. https://www.newsguardtech.com/special-reports/israel-hamas-war-misinformation-tracking-center/. Accessed 23 March 2024.

[36] G. S. Jowett, V. O’Donnell, Propaganda & Persuasion (Sage publications, 2018).

[37] D. Kellner, Media propaganda and spectacle in the war on Iraq: A critique of US broadcasting networks. Cult Stud.: Crit. Method **4**, 329–338 (2004).

[38] M. Pfau , Embedding journalists in military combat units: Impact on newspaper story frames and tone. J. Mass Commun. Q. **81**, 74–88 (2004).

[39] A. P. Cortell, R. M. Eisinger, S. L. Althaus, Why embed? Explaining the Bush Administration’s decision to embed reporters in the 2003 invasion of Iraq. Am. Behav. Sci. **52**, 657–677 (2009).

[40] A. Fichera, The horrifying images are real. But they’re not from the Israel-Gaza War. New York Times, 2 November 2023. https://www.nytimes.com/2023/11/02/us/politics/israel-gaza-war-misinformation-videos.html. Accessed 18 November 2023.

[41] R. Pogrebin, At a Hollywood screening, footage of Hamas killing Israelis. New York Times, 9 November 2023. https://www.nytimes.com/2023/11/09/arts/screening-footage-israel-hollywood.html. Accessed 18 November 2023.

[42] I. D. Cohen, How Israeli media became a wartime government propaganda arm. Haaretz, 25 December 2023. https://www.haaretz.com/israel-news/2023-12-25/ty-article-magazine/.premium/how-israeli-media-became-a-wartime-government-propaganda-arm/0000018c-a0d3-d957-a98f-aed3ea560000. Accessed 3 March 2024.

[43] S. Sontag, On Photography (Macmillan, 1977).

[44] M. Fischer, N. Nix, Raw videos of violent incidents in Texas rekindle debate about graphic images, Washington Post. 14 May 2023. https://www.washingtonpost.com/nation/2023/05/14/violence-graphic-images-texas/. Accessed 5 November 2023.

[45] S. Sontag, Looking at war. The New Yorker, 9 December 2002. https://www.newyorker.com/magazine/2002/12/09/looking-at-war. Accessed 5 November 2023.

[46] B. Zelizer, Finding aids to the past: Bearing personal witness to traumatic public events. Med. Cult. Soc. **24**, 697–714 (2002).

[47] D. Harwell, Violent videos and “brutal voyeurism” are redefining modern war. Washington Post, 24 October 2023. https://www.washingtonpost.com/technology/2023/10/24/israel-war-violent-videos/. Accessed 18 November 2023.

[48] K. Collins, Why Israel showed searing images of Hamas attack. CNN, 23 October 2023. https://www.cnn.com/2023/10/29/world/why-israel-showed-searing-images-of-the-hamas-attack/index.html. Accessed 18 November 2023.

[49] A. Iyer, J. Webster, M. J. Hornsey, E. J. Vanman, Understanding the power of the picture: The effect of image content on emotional and political responses to terrorism. J. Appl. Soc. Psychol. **44**, 511–521 (2014).

[50] A. Geis, G. Schlag, ‘The facts cannot be denied’: Legitimacy, war and the use of chemical weapons in Syria. Global Discourse **7**, 285–303 (2017), 10.1080/23269995.2017.1288488.

[51] C. Tabachnick, State Department issues “worldwide caution” alert for U.S. citizens due to Israel-Hamas War. CBS News, 19 October 2023. https://www.cbsnews.com/news/state-department-worldwide-caution-alert-us-citizens-israel-hamas-war/. Accessed 5 November 2023.

[52] C. Archetti, Understanding Terrorism in the Age of Global Media: A Communication Approach (Springer, 2013).

[53] L. Polgreen, V. Darbha, News organizations should publish this photo. New York Times, 15 November 2023. https://www.nytimes.com/2023/11/15/opinion/gaza-war-children-image.html. Accessed 3 March 2024.

[54] M. M. Grynbaum, K. Robertson, Harsh visuals of war leave newsrooms facing tough choices. New York Times, 14 November 2023. https://www.nytimes.com/2023/11/14/business/media/israel-hamas-media-photography.html. Accessed 3 March 2024.

[55] O. Knox, C. Anders, A look inside The Post’s decision to publish photos from mass shootings. Washington Post, 16 November 2023. https://www.washingtonpost.com/politics/2023/11/16/look-inside-posts-decision-publish-photos-mass-shootings/. Accessed 3 March 2024.

[56] J. B. Houston, M. L. Spialek, J. First, Disaster media effects: A systematic review and synthesis based on the differential susceptibility to media effects model. J. Commun. **68**, 734–757 (2018).

[57] D. P. Relihan, K. D. Estes, D. R. Garfin, E. A. Holman, R. C. Silver, “Media images matter: Responses to Ukraine war media in a U.S. nationally representative sample”. Paper Presented at the Annual Meeting of the Society for Personality and Social Psychology (San Diego, CA, 2024), (February 2024).

[58] K. D. Estes, D. R. Garfin, E. A. Holman, R. C. Silver, Consuming hurricane-related media: The protective role of perceived trust. Psych. Trauma: Theory Res. Pract. Pol., in press. 10.1037/tra0001802 39509225

[59] A. Koenig, A. Lampros, Graphic: Trauma and Meaning in Our Online Lives (Cambridge University Press, 2023), 10.1017/9781108999687.

[60] J. G. Webster, The Marketplace of Attention: How Audiences Take Shape in a Digital Age (MIT Press, 2014).

[61] Federal Communications Commission, Obscene, indecent and profane broadcasts (2024). https://www.fcc.gov/consumers/guides/obscene-indecent-and-profane-broadcasts. Accessed 3 March 2024.

[62] E. Lempinen, Images of war are shocking. They can also strengthen our humanity. Berkeley News, 10 January 2024. https://news.berkeley.edu/2024/01/10/images-of-war-are-shocking-they-also-can-strengthen-our-humanity. Accessed 3 March 2024.

[63] J. W. Brehm, A Theory of Psychological Reactance (Academic Press, 1966).

[64] G. Russo, L. Verginer, M. H. Ribeiro, G. Casiraghi, “Spillover of antisocial behavior from fringe platforms: The unintended consequences of community banning” in Proceedings of the Seventeenth International AAAI Conference on Web and Social Media (Association for the Advancement of Artificial Intelligence, 2023).

[65] American Psychological Association, “Health advisory on social media use in adolescence” (American Psychological Association, 2023). May 2023.

[66] S.-H. Jeong, H. Cho, Y. Hwang, Media literacy interventions: A meta-analytic review. J. Commun. **62**, 454–472 (2012). 22736807 10.1111/j.1460-2466.2012.01643.x PMC3377317

[67] J. McDougall, K. Fowler-Watt, L. Edwards, Media literacy in the time of Covid. Sociol. Comunicazione **62**, 50–68 (2021).

